# “Bad boys don't cry”: a thematic analysis of interpersonal dynamics in interview narratives of young offenders with psychopathic traits

**DOI:** 10.3389/fpsyg.2015.00960

**Published:** 2015-07-07

**Authors:** Julie De Ganck, Stijn Vanheule

**Affiliations:** Department of Psychoanalysis and Clinical Consulting, Ghent UniversityGhent, Belgium

**Keywords:** psychopathy, juvenile delinquents, interpersonal relationships, talking therapy, self-protective factors, transference, psychoanalysis

## Abstract

Most discussions of the social and interpersonal styles in individuals with strong psychopathic traits focus on their dangerousness or their affective and interpersonal deficiencies. This study has a different focus, and starts from the idea that such focus on the threat emanating from individuals with a psychopathic style might blind us from the logic inherent to their way of relating with the world. By means of a qualitative analysis (thematic analysis) of narratives from a Lacanian talking therapy, this study examines how 15 youngsters with strong psychopathic traits make sense of interpersonal events and relations. The main recurring theme across these narratives was that others in general are fundamentally distrustful antagonists that they have to protect themselves from. Especially the father figure, with whom identification seems to take place, is seen as a violent actor. Consequently, these youngsters develop multiple strategies of dealing with the threat they experience in relation to (significant) others. These relationship patterns also emerged within the therapeutic relationship, resulting in frequent testing of the therapist's trustworthiness. The results of this study, discussed in terms of Lacanian theory, might help therapists to develop treatment approaches that better fit with the interpersonal orientation of individuals with strong psychopathic traits.

## Introduction

“With hard men intimacy is a thing of shame- and something precious.”

—Friedrich Nietzsche, Beyond Good and Evil

In recent decades scientific interest in the concept of psychopathy has strongly increased. Currently, most studies start from Hare's (2003, 2011) model, which defines psychopathy as a severe and stable disorder that consists of four dimensions: (1) an arrogant, deceitful *interpersonal* style, (2) a defective *affective* life, (3) an impulsive-irresponsible, and (4) socially deviant *lifestyle*. At the *interpersonal* level, psychopaths are considered to be glib-tongued, superficial, narcissistic, grandiose, egocentric, deceptive, and manipulating (Hare, 2003; Hare and Neumann, 2008, 2009). Meloy (1988) and Hare (2011) describe the reptilian-like and predatory gaze of the psychopath that leaves most people uncomfortable, almost as if they feel like potential prey in the presence of a predator: “*Psychopaths* are social predators who charm, manipulate, and ruthlessly plow their way through life, leaving a broad trail of broken hearts, shattered expectations, and empty wallets. Completely lacking in conscience and in feelings for others, they selfishly take what they want and do as they please, violating social norms and expectations without the slightest sense of guilt or regret” (Hare, 2011, p. 200). These interpersonal characteristics are often connected with a *socially aberrant way of living*, marked by an excessive need for excitement and impulsive, irresponsible, and rule-violating behavior. Their lack of empathy, their incapacity for close relationships, together with their grandiosity, and egocentricity might pave the way for antisocial and criminal behavior (Porter, 2007; Hare and Neumann, 2009). Indeed, psychopathy is a strong risk factor for antisocial conduct, institutional maladjustment, recidivism, and violence (e.g., Hare, 2006; Hare and Neumann, 2009). Research on psychopathy in minors indicates that these interpersonal and anti-social traits can be observed in young people as well as adults (e.g., Vasey et al., 2005).

The concept of child or juvenile psychopathy has a long history. For example, in his seminal text, *The mask of Sanity*, Cleckley (1976) suggested that psychopathy has its roots in childhood. Also McCord and McCord (1956) contended that the child psychopath has the embryonic personality traits (i.e., a lack of anxiety, lack of identifying ability, and a lack of guilt) of adult psychopathy: “His tantrums and delinquencies betray his aggressiveness. His truancies reflect his impulsivity. His cruelties to animals and children reveal his asociality. The child psychopath has little if any—remorse for his diffuse, brutal, usually purposeless activities, and he seems unable to affiliate with other human beings” (p. 99). Since the 1990s, much research has investigated whether juvenile psychopathy can indeed be defined by the same constellation of traits as its adult counterpart, and whether it is surrounded by a nomological network similar to that of adult psychopathy (Salekin and Lynam, 2010). The development of the PCL-R (Hare, 1991) revitalized the research into child and juvenile psychopathy. In particular Lynam (1996, 1997, 1998) and Frick et al. (1994), made great efforts to extend the construct of psychopathy to youth and to “capture the fledgling psychopath in a nomological net.” Frick et al. (1994) took on the task of validating the construct of child psychopathy by focusing on the presence of callous and unemotional traits (e.g., lack of remorse and empathy). Factor analysis of their newly developed Psychopathy Screening Device (Frick et al., 1994) in a sample of 95 clinically referred children generated two factors; a Callous Unemotional (CU) factor and an Impulsive Conduct Problems (ICP) factor. According to Frick et al. (1994) the CU and ICP factors corresponded with the two factors found on the PCL-R (Hare, 1991). Subsequent studies (e.g., Bary et al., 2000; Frick and Marsee, 2006) indicated that CU traits are decisive for the identification of high-risk groups of antisocial youth, and suggest that conduct disordered youth with CU traits exhibit a range of features consistent with adult psychopathy. Lynam (1997, 1998) elaborated on Frick et al. (1994) work of validating the construct of child psychopathy and concluded that children who combined symptoms of hyperactivity-impulsivity-attention problems and conduct problems most closely resemble psychopathic adults. Based on a systematic construct validation study with his Child Psychopathy Scale, Lynam (1997) contended that childhood psychopathy fits into the nomological network surrounding adult psychopathy and that children with psychopathic traits, like adult psychopaths, were serious and stable offenders; impulsive; and more prone to externalizing than to internalizing psychopathology. With respect to externalizing problem behaviors, several studies with community and referred samples have indicated that juveniles with psychopathic traits share many features with adult psychopaths, such as more persistent, serious and violent antisocial behavior, and an increased risk of recidivism and institutional infractions (e.g., Toupin et al., 1996; Brandt et al., 1997; Stafford and Cornell, 2003; Corrado et al., 2004; Salekin et al., 2005).

However, such an excessive focus on the dangerousness and social inadequacy of individuals with psychopathic traits might undermine our understanding of their psychological and social functioning. By focusing on affective and interpersonal deficiencies we potentially lose sight of the logic inherent to their way of relating with the world.

Given their egocentricity and their problems with empathy and close relationships, psychotherapy with individuals with a psychopathic personality profile is often deemed impossible (e.g., Wong, 2000; Skeem et al., 2002; Harris and Rice, 2007). Some studies indicate that this kind of therapeutic defeatism may not be grounded, particularly in the context of young people (e.g., Salekin, 2002; McGauley et al., 2007; Polaschek and Daly, 2013). Indeed, psychopathic traits can change through psychotherapy, and the quality of the therapeutic relationship might facilitate this change (Chakhssi et al., 2014).

In line with psychotherapy researchers' observations that the effectiveness of treatment is strongly determined by the quality of the therapeutic relationship (e.g., Luborsky et al., 1985; Konzag et al., 2004), we believe that further research is needed into how individuals with psychopathic traits actually experience their interpersonal world. Qualitative investigations of how *they* make sense of interpersonal events might help therapists to develop treatment approaches that better fit with the interpersonal orientation of individuals with a psychopathic profile. Compared to the amount of studies using descriptive approaches of juvenile delinquents with psychopathic traits, very few studies explore these youngsters' first person perspective on what they live through. Indeed, a search on the Web of Science database using the terms “psychopathy” and “qualitative” revealed that while the need for such work has been noted (Sullivan, 2005), apart from case studies, qualitative research is lacking in contemporary literature. We believe that further insight into how juvenile delinquents with psychopathic traits make sense of themselves and others, and a good comprehension of the logic with which they approach the world, would provide invaluable background knowledge for psychotherapeutic interventions.

This study aims to explore how juvenile delinquents with psychopathic traits experience interpersonal relations, and how intersubjective dynamics are re-enacted in a therapeutic setting. We address three interrelated research questions:

- How do (significant) others emerge within the narratives of adolescents with psychopathic traits?
- Which modes of functioning and interrelating do these adolescents use in dealing with (significant) others?
- Which relationship patterns emerge within the therapeutic setting?

We address these questions in an explorative qualitative research design, focusing on therapeutic interview narratives of 15 adolescents. All participants have high scores on a frequently used assessment instrument for psychopathy: the Psychopathy Checklist: Youth Version (PCL:YV; Forth et al., 2003). The data are analyzed by means of thematic analysis.

## Methods

All participants were recruited from a Belgian 6-month residential treatment program for juvenile delinquents between the age of 14 and 17. The institution works with Multidimensional Family Therapy and aims to help young people live a crime-free life and generally integrate into society. Overall, the interventions aim to introduce structure and control in these youngsters' lives. The average duration of the treatment is 6 months. Individual psychotherapy is generally not part of the treatment program.

From a total sample of 42 male juvenile delinquents, all of whom were also involved in a broader research project on psychopathic traits in adolescent delinquents, we first selected the adolescents with a high score (i.e., a score of 30 or more) on the PCL:YV (*M* = 31.9; *SD* = 1.7, Forth et al., 2003). Fifteen adolescents were selected. The PCL: YV rating is based on a semi-structured interview and file-data. It consists of 20 items that are scored on a three-point ordinal scale: definitely not present (0), partially present (1), or definitely present (2). The total score, ranging from 0 to 40, reflects the degree of psychopathy. All participants lived in Belgium. As the sessions progressed it became clear that some youngsters lived in intact families, while others did not. However, the current family constellation was not systematically mapped for the study participants. Also, sexual orientation of participants and their parents was not recorded.

In a second step these 15 youngsters were invited to engage in talking therapy, focusing on the problems they experience in their lives. It was explained that all sessions would be conducted by the first author, who is a trained psychoanalytic therapist (focus on Lacanian psychoanalysis). Participants were informed that all sessions would be audiotaped and transcribed, in order to be studied at a later stage. All participants gave their informed consent. The mean age of participants was 15.3 years (*SD* = 1.1). All sessions took place in the institution. On average participants engaged in 10.2 sessions (*SD* = 6.7).

It is important to note that none of these talking therapies were initiated by a direct demand from the participant. All individuals participated following the therapist's invitation. Before the request for engagement in a talking therapy was formulated, the youngsters were familiarized with the therapist, as she worked as a participant observer in the institution for several months, and also made a documentary film with a number of them. While all agreed to explore the problems they experienced, the exact reasons as to why they wished to participate were not recorded. Consequently, none of the youngsters entered therapy with a clear demand or complaint. However, as therapy progressed some developed a true analytic demand, meaning that they connected an element of personal suffering to the question of how they were involved in their own problems (Zenoni, 1989). Indeed, after the project in the institution came to an end, some youngsters contacted the therapist with a request for further sessions, in order to question aspects of their own functioning.

The research project was approved by the Ethics Committee of the Faculty of Psychology and Educational Sciences at Ghent University.

In conducting the talking therapy the therapist did not hold to a treatment protocol. In line with her training in Lacanian psychoanalytic therapy, she invited the participating youngsters to talk about their concerns in life. In working with their stories she adhered to the principles and techniques of a Lacanian psychoanalytic intervention (Fink, 2007). As is usually the case in Lacanian psychoanalytic therapy, no formal psychological assessment procedures were applied prior to the commencement of the therapeutic sessions, which focused on the adolescents' experiences and difficulties.

All sessions were audio-recorded and transcribed verbatim. The interview narratives were analyzed by means of *thematic analysis*. “Thematic analysis is a method for identifying, analysing and reporting patterns (themes) within data. It minimally organizes and describes your data set in (rich) detail” (Braun and Clarke, 2006, p. 79). In interpreting the data we were guided by our theoretical background in Lacanian psychoanalysis. Thematic analysis “is not wedded to any pre-existing theoretical framework, and therefore it can be used within different theoretical frameworks” (Braun and Clarke, 2006, p. 81). In the context of the present study, we took into account our broader knowledge of literature on psychopathy. However, in order to avoid any biased interpretations or selectively focusing on particular fragments of sessions, we closely followed the thematic analysis guidelines of Braun and Clarke (2006). This six-step approach consists of: (1) Familiarizing yourself with your data; (2) Generating initial codes; (3) Searching for themes; (4) Reviewing themes; (5) Defining and naming themes; and (6) Producing the report.

We familiarized ourselves with the data (1) by transcribing the sessions and by controlling the exactness of the transcriptions thus obtained. The transcription process was assisted by MA students in psychology at Ghent University. In order to generate initial codes (2) the first author identified all narratives of interpersonal encounters, descriptions of significant others and interpersonal dynamics within the therapeutic setting. As these sections were selected we also briefly noted what exactly was being discussed. Subsequently, the authors located and discussed recurring patterns of the interpersonal relations across all participants, thus identifying overarching themes in the data (3). A list of themes were identified, which were then discussed and grouped into clustered themes, largely corresponding to the themes in the Result Section below (4). In doing so we compared data-extracts across participants and aimed to detect diverse narratives corresponding to the overall themes. The authors agreed on the topics each theme consists of and highlighted illustrative examples. Consensus regarding the main themes and topics, and concerning illustrative examples was reached between the authors. In a next step (5) the exact wording of each theme and its topics was discussed. The outcome of these discussions comprise the Results Section of this paper (6).

For the purpose of checking the reliability of our results, a student completing an MA dissertation on these data independently conducted a thematic analysis on all session narratives of six participants of the study. Similar themes were identified, which brought us to conclude that no additional categories needed to be created.

## Results

### Who is the other to them?

Common descriptions of individuals with psychopathic traits often stress that they are fearless and hostile, depicting the psychopath as a social predator (Meloy, 1988) or a bull terrier (Lykken, 1995). According to this assumption, we are at risk of falling prey to the psychopath's malevolent intentions. However, as our therapeutic sessions indicate, in the view of our participants the opposite is true. Indeed, the predominant theme recurring across the narratives of all 15 adolescents was that it is *they* who tend to fall prey to others: (significant) others are *fundamentally distrustful* antagonists that they must guard themselves from. This is illustrated by Lukas (session 10):

Never underestimate a man and never give a man your trust. Wait and see (…) If you know someone for 10 years, then you can tell him about 10% about yourself. Then, you observe. And if you can really (…) feel his heart, his soul… (…) then you can tell him another 30%. (…) There are people who'll immediately tell you everything about themselves. Then there's deception. They loved a girl, trusted her, and in retrospect they see she was actually a little whore. (…) And you too (…): “You should not trust anyone, miss.”

Throughout the interviews, this “threatening other” proved to be embodied in three different character types: (a) the malignant other; (b) the annoyingly different other; and (c) the taunting other. Below we describe these three characters and discuss how the maternal and paternal figures are frequently depicted in our participants' narratives of intersubjective relationships.

#### The malignant other

The figure of an enigmatic, incomprehensible and threatening other was predominant in all participants' stories. As they fail to unravel what the other wants from them, basic distrust prevails (Vanheule, 2011): the other is not seen as a partner they can rely upon, but as a figure they are subjected to. Often, evil is perceived in the other's *gaze*, as illustrated by Bastian (session 5):

I often lose control and get angry. (…) when someone looks at me with an evil gaze. Then you know his intentions are malicious, that he wants a fight. (…) Then everything turns black before my eyes (…). I lose control, I fight, (…) or destroy things. (…) It's the wicked gaze of the other.

Moreover, frequently *a demanding other who comes uncomfortably close* was experienced as malevolent. All participants expressed difficulties in enduring intimate relationships, both with family members and (girl) friends. This seems to result from an inability to decode others' motives and a fear of losing control, as illustrated in the following fragments:

I've never been in love. Being in love… (…) I would not be able to stand it. (…) I would go crazy, lose control. Girls, they make you crazy. I couldn't stand the idea that I'd always want to be with her (Dennis, session 7).

I don't know from what kind of mother and father I descend. (…) They are not the kind of people to mess with. (…) A man or a thief or an animal… An animal, when it's hungry it goes to its mother, right? To his own mother, not to cows or monkeys, right? A cow has a baby and the baby knows his mother because he needs to eat. This mother goes to the child, to stay close to the child, to give it warmth, to defend it (…). But this mother comes too close for comfort. The child needs freedom. So he has to go. He has to leave his mother, (…) because an animal knows who his mother is. I know who my mother is (Max, session 16).

Others are typically seen as violent deceivers to which they could fall victim; participants don't assume that intersubjective relationships are regulated by social rules that safeguard those involved. In their view, words and laws are deceptive, which is why nothing coming from the other is taken for granted, as illustrated by David (session 7):

People (…) cheat, they're hypocritical, their word is not to be trusted. (…). In this world… no-one is perfect. Everyone… even teachers, bank managers… they snort coke for example. You may not be aware of that (…) I mean… You think: that's a teacher, that's a doctor, a bank manager, a respectable man…(…) who works for a living, has a family… But then, you see them using coke, you know they're violent at home… (…) so you see how banks get robbed… The thieves know exactly (…) where the money is… They're tipped, miss, by those perfect men.

#### The annoyingly different other and the importance of the ideal-ego

While they do not have confidence in social rules and laws, they maintain a relation to others through identification with an extremely masculine and aggressive ideal-ego. By means of this virile and hostile imago they can transcend their experience of fear, as illustrated by Jonas (session 1);

I'm a cold-blooded human being, just like my father, like my entire family. I once ate a hedgehog… its liver, its heart (…). I drank its blood and ate its flesh. Since that moment, I'm a cold-blooded man. I like to see blood. Since I drank its blood I'm cold-blooded and without fear. (…) Some people are always afraid, I'm not, I always laugh.

The identification with this virile and hostile imago gives them a stable sense of identity, a sense of *being* someone. However, this identification is fragile and is challenged in every encounter with another person who differs in some way (e.g., appearance, dealing with emotions, etc.) from this ideal-ego. As they don't believe that interpersonal relationships are regulated by social rules, a confrontation with the “otherness of the other” (Lacan, 1966 [2006]) proves to be threatening or frustrating. This often results in aggression because the ego is threatened, as illustrated by Dennis (session 5), who strongly adheres to the imago of “the bad guy that never cries”:

D.: I've never had that feeling of being sad or…(…) They can't hurt me or destroy me. Nobody, no Judge can break me, you must let them know this (…). I hate misery and people acting hopelessly… I hate it when people around me cry. I say “Shut up!”

J.: Other people crying is unbearable for you?

D.: I hate that, I get angry when they act hopelessly. Act like a man! A man doesn't cry.

J.: Who says a man doesn't cry?

D.: I do, I say to them: “A man does not cry, so why are you crying?” (…) Life is hard, so you have to be hard.

#### The taunting other and the narcissistic injury

The character of the taunting other concerns an individual that literally insults the adolescent's ideal-ego, or who offends one of the significant others from his life, particularly the mother. This seems to produce an accumulation of tension and anger, due to the discrepancy arising between the ideal-ego, or the ideal image of the (m)other, and produces an experience of narcissistic humiliation (Baumeister et al., 1996), as illustrated by Max (session 16):

I don't know how to control myself. (…) People will provoke you. They'll say: “Your mother (…) is a faggot or a whore” (chuckle). They call me a loser. At school they do. The teachers do. (…) I can 't take it. I lose control.

### The paternal and maternal other

Particular parent-child relationship patterns recurred with considerable frequency in the session narratives of our participants. At first sight, basic distrust is not experienced toward the paternal and maternal other: e.g., “My family has made a great man of me, of course I trust them” (Lukas, session 5). Moreover, several adolescents in our study differentiate between their lives as delinquents in the outside world (which is conceived of as dangerous) and their lives as a son within their families (which is perceived as safe and reliable): e.g., “At home I trust everybody, but as soon as I'm in the outside world, everything is different” (Caspar, session 12). The paternal other is often described as a righteous, respectable, intelligent, but frequently gadabout man. The maternal other is often idealized as a kind of holy Madonna figure, as illustrated by Dennis and Jonas:

My father means a lot to me. He's… someone important. A smart person, yes. Not an aggressor, a quiet man. He loves nature and jazz music (…) and opera. He listens to this kind of music when he's reading. (…) He's not violent. He doesn't use swear words against anyone. He's a proper man (Dennis, session 10).

However, a process of dissociation with regard to the image of the paternal other could be observed in most of the participants. For example, the image Dennis (session 10) outlines of his father is strongly contradicted by later statements describing his father as an aggressor:

If I did something wrong as a child, my father would try to scare me by saying the police were coming to arrest me. It didn't bother me. Then he'd physically assault me or break my bones. (…) Then I got beaten up by him and I had to sleep in the basement as a punishment. I didn't understand that. (…) He was just angry because I used to fight and extort. (…) He'd say: “You shouldn't think no-one sees you, Dennis. You damage my good name” (Dennis, session 10).

Initially these different images of the parental other are dissociated from one another. Yet, we observed that in the end they are integrated, with the adolescent believing in the legitimacy of violent and brutal actions: “How I feel when I got hit by my father? I deserve it. You get what you deserve. That's the way it goes.” The father figure is not subjected to conventions and laws, but a capricious figure who imposes his will and whims onto others. Accordingly, the idealized image of the mother is frequently brought down by a violent paternal figure, as illustrated by Max (session 6):

In my family they never use violence, never! (…) Only 2 or 3 years ago, my father put a knife in my mother's neck (…) 6 cm deep or so. (…) It was dinnertime. My mother was (…) teasing my father. He got angry. (…) He didn't bring her to the hospital, he left her like that. (…) It doesn't matter, miss. (…) I said to my mother: “You shouldn't disturb dad when he comes home from work. He's a lot on his mind.” She shouldn't nag him. I think my father was right. (…). If she was looking for trouble, then you can get trouble, right? I hate that too when people interrupt me when I am eating.

These examples illustrate that probably the identification with an aggressive ideal-ego is rooted in an identification with the image of the paternal other, as is also illustrated by Lukas (session 10):

Our culture passes it on… (…) If my father's a thief, I'm a thief. If my father's a businessman, I'm a businessman. If my mother's a whore, my sister's a whore. (…) I'm not saying my family are thieves or whores. (…) It amounts to that. The father teaches you whatever (…).

### How do they deal with the other?

The participants indicated that they developed several self-protective strategies to deal with the unreliable and hostile world they experience. We observed four recurring strategies to deal with the threatening other they feel confronted with, which we discuss below.

#### Testing the reliability of the other, Hic Et Nunc

A thorough examination of how others behave and deal with confidential information is a commonly used strategy for testing the reliability of the other, as illustrated by Lukas (session 10):

Your heart is like a testament. You see and hear things, you observe people and then you can make a decision with your heart (…): “Yes, I can trust this person.” (…) Sometimes you really don't know whether you can trust your heart or mind. So you stage things to see what will happen. (…) For example, you have your car keys in your pocket, but you say to your friend: “Oh no, I lost the keys of my new BMW.” Then you leave your wallet with 1000 euros in it on the table, and watch what happens to your wallet when you go out looking for your keys.

Examples of this strategy were multiple. Some participants told that they organized meetings with members of the gang they belong to in order to check everyone's judicial declaration and to assess who is trustworthy and who is not. Others said that they told a secret to a friend and then observed whether that friend kept the secret or not, etc.

#### Acting as an outlaw

Another mechanism for escaping the menacing other consists of demonstrating that a nonviolent law guaranteeing safety doesn't exist. By acting as an outlaw, they seem to be demonstrating that the world is just a place of pretense and appearances. On the one hand, they denounce the semblance in the world by repeatedly challenging and provoking (representatives) of the law. For example, some participants said that they would race past the police station in a stolen car, play extremely loud music, or break into people's houses in broad daylight, such that passers-by could see them. Along that way they seem to be demonstrating that the law is not effective in regulating community life.

On the other hand, they demonstrated the absence of a guaranteeing law by inducing fear in the other. When anxiety is expressed in the other, they themselves seem to be able to overcome their own fears and manifest themselves as a subject in an interpersonal scene. Alexander's narratives illustrate this (session 8):

When you do a robbery… it feels great. (…) First you're standing outside. I always listen to music first, otherwise I won't go in. (…) I lift myself in. (…) While I'm changing clothes outside, another boy goes in and asks the pharmacist for something from the back (…) and then we go in. (…) At that moment, outside, I feel stressed. (…) Then we're laughing. (…) I've got music in my ears and I'm singing along. I'm getting charged up. And then I go in. (…) Then I start to laugh, with a little bit of stress (chuckles), a little panic. (…) It just feels good. I don't know why. (…) The woman is in the back. When she returns and she sees us, she's gonna be scared, afraid. (…) That's the best feeling (laughing). Sorry…

#### Testing the sameness—otherness of the other

As described above: the confrontation with the “otherness of the other” threatens the ego. Therefore, several of the participants engage in testing *the sameness or otherness* of the other. The question they implicitly seem to be addressing goes as follows: “Are you like me?” If the other acts according to their own ideal image, they conclude that the other is reliable. Along this way, they say that often friendships are created through fights or by committing crimes together, as illustrated by Dennis and David:

From childhood onwards (…) I beat up children when they asked me if I would be their friend. I kicked them in the face. (…) Some of them would cry, others wouldn't. Those who were able to endure the beating could enter my group. (Dennis, session 5). We met each other during a burglary. (…) We didn't know each other. We didn't wait until the streets were empty to break into houses. Showing off. He wasn't afraid of anyone. (…) After this burglary, we were always together, inseparable. Committing crimes together. (…) We didn't lack anything. (…) To trust someone means having no shortages (David, session 7).

#### Destruction of the other

*Destructing the other*, whether literally or not, is the final mechanism in place to protect against a deceiving other. Several fragments above already illustrate such use of violence: when the participant feels narcissistically hurt he attacks the other. Another strategy is to live the life of a *lone wolf*: “No, I don't need any friends. I prefer to live on my own. I don't like to make new friends. (…) Then you have to learn to trust all those suckers again (…). I don't like to trust people” (Thomas, session 1). Several participants seem engaged in doing everything in their power to not become emotionally dependent on someone. Some said that they radically ended (love) relationships out of fear of what love may bring:

Being alone in this world. I think it's better to be on your own. (…) I often retreat, away from other people. (…) Taking care of my own. In life, I only loved my sister who died. She was blind, (….) She had a tumor in her eye. (…) I often pulled her leg. (…) when she walked down the hallway, I'd sneak up behind her without saying anything. She could feel my presence and ask if someone was there. I never answered and I knew she was scared (David, session 7).

### How does the other emerge within therapy?

In a third step, we examined how these youngsters behaved within the context of the talking therapy, taking into account their overall image of the other and their habitual ways of dealing with the other. We observed that as they entered the therapeutic relationship the figure of the malignant other played a predominant role. Several participants had difficulties keeping eye contact [e.g., “your stare is weird, stop looking at me” (David, session 1)], or tolerate the closeness that the therapeutic process often entails. Clinging to their image of impenetrability, questions about emotional pain and fear were often deflected by the participants, especially in early sessions. For example, in response to the interviewer's question as to whether he had ever lost a friend in a fight, Dennis (session 12) answers: “No, no (hesitating)… It doesn't matter to me (sigh), miss. I am not used to talking about myself. It doesn't matter. Let's move on to the next question.”

Yet, as the sessions progressed it became clear that the participants were not so much distant because of a mere absence of (negative) affects. It rather seemed that they lacked the skills to cope with strong emotional experiences in interpersonal relationships. For example, in later sessions Dennis talked about how he had lost some friends, indicating that he didn't know any other way of dealing with it than to deny his sadness and become angry and frustrated. Later he asked the therapist if she had ever lost someone and if she could teach him different ways of dealing with grief and loss. Disclosure about their affective life was also inhibited by difficulties trusting the therapist. Given their habitual distrust in the other, most of them explicitly tested the confidentiality of the sessions. Especially in early sessions, several strategies were used to test the trustworthiness of the interviewer and to keep her at a safe distance. For example, they tested how she dealt with confidential information, as illustrated by Max (session 12):

M.: So you say I can trust you miss. It's not like that it'll suddenly emerge that you're not to be trusted? (…)

*-Max puts his cell phone on the table in the therapy room, while residents of the institution are not allowed to keep a cell phone with them*.

M.: Don't you have to ask me what my mobile is doing here? They didn't find it yesterday when there was a room inspection.

J.: (…) I would like to ask you something. Why do you tell me all these things?

M.: Every time when I come to you I remember what you said in the beginning, that all will stay confidential, that I can trust you. (…)

J.: But why do you want to show me that you smuggled in your mobile?

M.: Because you told me I can trust you. (…)

J.: And now you want to test whether I'm a person you can trust?

M.: Yeah. If they discover that I have my mobile, then I know it comes from you, Julie. (laughing) It's not something to laugh about. I'm bloody serious.

Another strategy for investigating the therapist's motives was by provoking the arbitrariness of professional secrecy and a guaranteeing law, as illustrated by Alexander (session 11):

*During the session the adolescent is toying with a broken pen. With the sharp point he is continuously reaching toward the wall, just nearly missing it*.

A.: What would you do if I were to smudge your beautiful new wall?

J.: Why would you do that?

A.: Seriously, miss, what would you do if I were to smudge the wall? Would you go and tell the principal?

J.: I think I would have to tell him, yes. The wall has just been painted.

A.: Is that so? You'd tell him, huh? Is my life in danger then? I thought you would only speak if my life or somebody else's life was in danger. That was the rule, I thought.

Taking control by fear-inducing or violent strategies occurred a few times, but was not dominant throughout the therapy, as illustrated in the following dialog with Lukas (session 10):

J.: But in court, they don't know you use a fake ID?

L.: Not when Julie doesn't tell anyone.

J.: I'm a psychologist, not a judge.

L.: Are you sure, Julie? (…) Are you sure you have only one key in your pocket? That of this institution?

J.: Do you think I also have the key to the courthouse?

L.: I've already checked everything. (…) Don't be afraid. If I were to tell you your address, where you live, when you were born, where your sisters live, would you be scared then?

The figures of the annoyingly different and taunting other hardly played any role within the therapeutic relationship. Only two fragments depict an adolescent feeling offended by the questions of the interviewer; “What do you think, miss, that I am a psychopath?” Only a few statements were related to the perception of “the otherness” of the interviewer; e.g., “Girls, like you,” “You can't understand that, miss, it's not your kind of world.” Occasionally, the sameness—otherness of the interviewer was examined, for example by asking her about her criminal background: “Miss, your boots… I know they are very expensive. How much did they cost, miss? I want to know. Where did you get them? On the black market? Illegally, right?” (Bastian, session 5) Initially, the otherness of the interviewer could be a source of frustration and threat, but became more accepted as sessions progressed in time.

As time progressed a positive therapeutic relationship was established with most participants. To a certain extent, they were willing to disclose most sensitive themes. In some adolescents a longing for a nonviolent way of *being* emerged, often combined with taking distance from the identification with a hard-hearted paternal figure:

My parents are dangerous, miss, especially my father. Give him a gun and he will shoot you. They don't reflect on what they do. (…) The moment he put the knife into my mother's back, I copied him. (…) But I'm not like my father (Max, session 16).

However, generally the therapeutic relationship remained fragile. For example, several adolescents asked for extra counseling sessions a long time after their discharge from the institution. For example, 1 year after his release from the institution, David contacted the first author because of depressive symptoms following the death of a friend. He asked her for one therapeutic session, and only one. It might be that by strictly limiting the encounter with the therapist in time, he aimed to escape from the position of vulnerability the therapeutic process put him in. The following session narrative also illustrates that even though there is an agreement of trust between adolescent and therapist it is often difficult for them to believe in the authenticity of words and relationships:

Our lives will separate here, miss. (…) I'll remember this conversation and I'll be happy. But I know that you'll go home and that you won't remember this evening. I know it's your job. I'm grateful that I could come (…). (David, session 7)

The moment the interviewer is about to express that these conversations are not without significance for her, he continues:

Miss, please don't say anything, don't say that you will remember this conversation (…) because then you're… It's fine as it is now…(David, session 7).

## Discussion

Most discussions of the social and interpersonal styles of individuals with strong psychopathic traits focus on their dangerousness in relation to others, or on their affective and interpersonal deficiencies. This study has taken a different focus, and starts from the idea that the usual focus on the threat emanating from individuals with a psychopathic style might blind us from the logic inherant to their way of relating with the world. Crude metaphors that compare psychopaths to predatory animals may guide professionals away from questions concerning how these people make sense of themselves and the world. More insight is needed into how individuals with strong psychopathic traits actually experience their interpersonal relationships and social dynamics. By means of a qualitative analysis (thematic analysis) of narratives from a Lacanian talking therapy, we examined how 15 youngsters with strong psychopathic traits make sense of interpersonal events and relations. This could help therapists develop treatment approaches that better fit with the interpersonal orientation of individuals with a psychopathic personality profile.

First we examined how (significant) others emerge within the narratives of these adolescents. The major recurring theme across the narratives of all 15 adolescents was that *they* tend to fall prey of others: others are *fundamentally distrustful* antagonists that they must protect themselves from. We observed that others are often seen as malignant deceivers, and as a result close relationships are poorly tolerated. We also observed that in relation to others they often profile themselves as virile and hostile individuals, often resulting in annoyance about people that are different from them. Insults from others have a dramatic impact and are frequently experienced as narcissistic humiliations. Finally, our participants bear witness of extremely violent father figures who impose their will and whims onto the world.

Next, we studied the modes of functioning and relating used by these adolescents in the context of the threat they experience as coming from (significant) others. Here we observed four recurrent strategies. First, they frequently engage in testing whether the other is reliable or not. Second, in order to escape from menacing others they attempt to demonstrate that a nonviolent law that guarantees safety doesn't exist. Testing the sameness or otherness of the other in relation to oneself was a third strategy we observed them applying in interpersonal relations. Fourth, we observed that interpersonal violence was often used to manage the threat experienced as coming from the other.

Finally we examined if and how these relationship patterns emerged within the therapeutic relationship. We observed that quite typically, as they entered the therapeutic relationship the figure of the malignant other played a predominant role. Distrust often stood to the fore, and relational closeness was avoided. Accordingly, the therapist's trustworthiness was often explicitly tested, for example, by checking whether she held her promise of confidentiality or by exploring how she related to the rules of the institution, as well as criminality in general. Trusting the therapist was not self-evident, meaning that trust had to be established time and again. As time progressed several participants came to disclose more and generally speak more openly about sensitive issues, sometimes resulting in distance taking from the harsh paternal figure. However, most commonly participants continued to struggle with the dilemma of such disclosure and experienced uncertainty around being in the hands of the therapist.

Overall, the participants' session narratives clearly indicate that much of their psychopathic actions are rooted in an underlying anxious and hostile interpretation of the social world, which is in line with findings from other studies (Serin, 1991; Vitale et al., 2005). Violence may function as a counter-reaction that helps them avoid a position of radical helplessness when feeling subjected to others that cannot be trusted (Vanheule and Hauser, 2008). Indeed, through case study material in another study, we demonstrate that the anxious and hostile interpretations of the social world described in this study often cohere with identifications with the image of “the criminal,” along which they position their ego in relation to perceived threats coming from without (De Ganck and Vanheule, 2015). We believe that the “mask of criminality” that youngsters with strong psychopathic traits often cultivate, and that frequently serves as a basis for the formation of gangs, makes up a masquerade via which the enigmatic but antagonistic other is kept at a safe distance.

Considered from the perspective of Lacanian theory, a safe symbolic law that guides human interaction seems missing for these young people. As a result, imaginary dynamics of aggressiveness dominate their interaction with others. In line with anthropologist Claude Lévi-Strauss, Lacan assumes that social groups, such as a family, have an underlying elementary structure, which consists of positions (e.g., mother—father—child) that function according to rules relating to what they can and can't do (Lévi-Strauss, 1949, 1958; Lacan, [1955–1956] 1993; Vanheule, 2011). Indeed, via language we attribute positions to individuals and at the same time unconsciously follow laws and rules of exchange. With this symbolic structure, the actions of others are, to an extent, predictable for the individual. The narratives collected in this study bear witness of the opposite, indicating that the social world of these youngsters does not appear to be structured in this way: other people within their social system *do not* appear to occupy clear positions or behave according to lawful principles. For this reason, other people's motives and desires emerge as *enigmas* they cannot be made sense of, rendering the world an extremely unpredictable place to live in. Indeed, no clear position can be attributed to father figures in particular, and no stable law seems to determine their actions. This undermines the experience of the symbolic order and opens up the realm of the psychotic experience, in which the subject has to deal with a “mad” other (Lacan, [1959] 2006; Regnault, 1995; Vanheule, 2011). When others get too close they are unpredictable, and by using violence a safe distance is recreated. This inability to endure intimate relationships in psychopathy was also observed by Vaillant(1975, p. 181) who states: “Close relationships arouse anxiety in them. Terrified of their own dependency, of their very “grievance,” and of their fantasies of mutual destruction they either flee relationships or destroy them.” To some extent, the extreme *identification with the image* of the “fearless criminal” enables them to position themself in relation to others. Radical identification with “aggressiveness” seems to provide them with the sense of *being* someone. Instead of being overwhelmed and intimidated by the enigma of the other, passing to the act enables them to proactively assert their identity. This identity qua criminal has both a separating and identity creating function: it enables them to keep the enigmatic (desire of the) other at a distance, and at the same time to create a feeling of being someone.

In his third seminar, as he discusses the problems of psychopathic delinquency in relation to psychosis, Lacan ([1955–1956] 1993, p. 204) suggests that in case of “psychopathic personality inversion” the subject is radically subjected to the other qua “social monster.” Father figures seem to function as radically cruel creatures, that are not guided by the pact, but impose their will onto the world. Lacan suggests that in relation to such another, only two possibilities remain open for the subject. Either he is completely intimidated and undergoes the regime of terror. Alternatively he might identify himself with the image of the social monster himself and thus try to create an equilibrium in relation to others that enter his world. The results of our study seem to underscore this logic.

Therefore, we believe that in the context of psychotherapeutic relations, psychopathic behavior should be thought of as a self-protective strategy for managing a fundamentally fearful position. Many therapies focus on eliminating psychopathic features and reducing the risk of recidivism. However, we argue that such change can only be obtained if the underlying anxiety and distrust is taken into account. We observed that these youngsters are not immune to the painful experiences of grief, fear and self-doubt. However, their basic distrust inhibits them in expressing emotions. Expressing private experiences tends to bring them to the mercy of the other that they distrust. Thus, the main task for the therapist consists in creating a safe therapeutic environment. For realizing such therapeutic environment, an attitude of neutrality, which is essential to all forms of psychoanalytic therapy, is crucial. We observed that actively guaranteeing professional confidentiality was a necessary (but not sufficient) condition to obtain minimal trust. After all, for these adolescents we, as therapists, are a menace; to them we represent a deceitful and threatening society. To protect them against danger, professional confidentiality might be tested, lies might be told, inner feelings might be masqueraded, and fear-inducing strategies might be used. We believe that this “testing” should be tolerated by the therapist. For example, when it became clear that one of our participants had lied, we did not show anger, and refrained from framing lying as a moral issue, but referred to the agreement that everything *could* be said within the therapy, including lies. Subsequently, we invited him to reflect on why it was necessary for him to lie. We also never put pressure on adolescents to talk about anything, including their criminal offenses. We stated from the outset that it was not the role of the therapist (in contrast to the police or Juvenile Court) to uncover the truth behind their criminal offenses and in that way they were allowed to withhold whatever information they wished. Anytime they spoke openly about criminal offenses or about violations of the rules in the institution, the therapist referred consistently to their act of violating the rule, but not in a judgmental way. What we consider as important in this is that the therapist behaved as an individual who was subject to conventional laws as well. For example, whenever she had violated certain social rules the therapist took personal responsibility, e.g., by recognizing her mistake if she showed up late for a session. It was partly due to these small but human(izing) interventions that a positive therapeutic relationship was established. To the extent that she was subjected to rules and adopted a non-moralizing attitude toward these youngsters, the therapist was a safe person to talk to.

In our opinion, one of the main obstacles to a successful therapeutic relationship with individuals with psychopath traits might be the fear of the therapist of being fooled by these patients. Lacan is quite radical on this matter, however: “There is only one resistance, the resistance of the analyst. The analyst resists when he doesn't understand what he is dealing with” (Lacan, [1954–1955] 1988, p. 228). To avoid such fears, an open and non-judgmental attitude on the part of the therapist is required, meaning that her fears need to be addressed in personal psychoanalysis and/or supervision. Accepting the psychological and interpersonal dynamics behind psychopathic behavior is of utmost importance. With this manuscript, we hope to have contributed to this perspective.

Nevertheless, there are some limitations to this study. First, on behalf of the interviews no other assessment instruments were used to explore participants' social and psychological functioning. Completing psychodynamic assessment of social and psychological functioning before the start of the interviews might have been relevant. This could have shed light on the participants' psychopathological organization. Second, this study specifically focused on experiences of adolescents with high psychopathy scores. The question as to whether and how these results might be generalized to adult populations cannot be answered based on our data. Future studies might focus on such comparative study. Our sample consisted of adolescents with high psychopathy scores. This implies that our results cannot easily be generalized to adults exceeding the treshold that is presumed to be indicative of psychopathy in psychopathy measurement instruments for adults. Third, this study might have important implications for how, at an institutional level, therapy for youngsters with strong psychopathic traits might be organized, which we have not discussed. Fourth, while during the sessions some participants discussed family-related problems in detail, we did not map the family constellation for each individual. This might be relevant for examining how particular ways of experiencing others are characteristic of specific family constellations. However, a particular experience of others was observed in the sessions with all youngsters. Fifth, we did not record the sexual orientation of participants and their parents, while this might have had an effect on how they relate to others. Sixth, our study is limited by the very short nature of the therapies (10.2 sessions on average). Follow-up studies that involve longer-term psychotherapies might be relevant for studying how transference evolves across time, and to explore if and how the testing behavior ever recedes.

### Conflict of interest statement

The authors declare that the research was conducted in the absence of any commercial or financial relationships that could be construed as a potential conflict of interest.
